# Accumulation of Extracellular Hyaluronan by Hyaluronan Synthase 3 Promotes Tumor Growth and Modulates the Pancreatic Cancer Microenvironment

**DOI:** 10.1155/2014/817613

**Published:** 2014-07-24

**Authors:** Anne Kultti, Chunmei Zhao, Netai C. Singha, Susan Zimmerman, Ryan J. Osgood, Rebecca Symons, Ping Jiang, Xiaoming Li, Curtis B. Thompson, Jeffrey R. Infante, Michael A. Jacobetz, David A. Tuveson, Gregory I. Frost, H. Michael Shepard, Zhongdong Huang

**Affiliations:** ^1^Halozyme Therapeutics, Inc., 11388 Sorrento Valley Road, San Diego, CA 92121, USA; ^2^Sarah Cannon Research Institute/Tennessee Oncology, PLLC, 250 25th Avenue North, Nashville, TN 37203, USA; ^3^Labfinity, 8110 Cordova Road, Suite 119, Cordova, TN 38016, USA; ^4^Cold Spring Harbor Laboratory, 1 Bungtown Road, Cold Spring Harbor, NY 11724, USA; ^5^Intrexon Corporation, 6620 Mesa Ridge Road, San Diego, CA 92121, USA

## Abstract

Extensive accumulation of the glycosaminoglycan hyaluronan is found in pancreatic cancer. The role of hyaluronan synthases 2 and 3 (HAS2, 3) was investigated in pancreatic cancer growth and the tumor microenvironment. Overexpression of HAS3 increased hyaluronan synthesis in BxPC-3 pancreatic cancer cells. In vivo, overexpression of HAS3 led to faster growing xenograft tumors with abundant extracellular hyaluronan accumulation. Treatment with pegylated human recombinant hyaluronidase (PEGPH20) removed extracellular hyaluronan and dramatically decreased the growth rate of BxPC-3 HAS3 tumors compared to parental tumors. PEGPH20 had a weaker effect on HAS2-overexpressing tumors which grew more slowly and contained both extracellular and intracellular hyaluronan. Accumulation of hyaluronan was associated with loss of plasma membrane E-cadherin and accumulation of cytoplasmic *β*-catenin, suggesting disruption of adherens junctions. PEGPH20 decreased the amount of nuclear hypoxia-related proteins and induced translocation of E-cadherin and *β*-catenin to the plasma membrane. Translocation of E-cadherin was also seen in tumors from a transgenic mouse model of pancreatic cancer and in a human non-small cell lung cancer sample from a patient treated with PEGPH20. In conclusion, hyaluronan accumulation by HAS3 favors pancreatic cancer growth, at least in part by decreasing epithelial cell adhesion, and PEGPH20 inhibits these changes and suppresses tumor growth.

## 1. Introduction

Pancreatic ductal adenocarcinoma is the fourth-leading cause of cancer-related deaths in the United States with a median 5-year survival rate of 6% [1]. Pancreatic cancer is characterized by a desmoplastic response involving stromal fibroblasts, inflammatory cells, and pathological deposition of altered extracellular matrix [2, 3] that contains high levels of fibrous collagen, proteoglycans, and glycosaminoglycans including hyaluronan (HA, hyaluronic acid) [3]. Hyaluronan is a negatively charged linear glycosaminoglycan composed of repeating disaccharide units of N-acetylglucosamine and D-glucuronic acid. Excess hyaluronan is found in several tumor types including pancreatic, breast, and prostate cancers [4–7] and its accumulation has been shown to be a factor associated with poor prognosis for cancer patients [5, 7, 8]. Hyaluronan accumulation leads to increased tumor interstitial fluid pressure and poor perfusion [6, 9] explained by continuous tumor cell secretion of hyaluronan and its substantial absorption of water molecules (~15 per disaccharide) [10]. Elevated tumor interstitial fluid pressure and poor perfusion can be normalized by hyaluronan depletion from the tumor [6, 9]. In addition, hypoxia, often found in advanced solid tumors and characterized by upregulation of hypoxia-inducible factor-1*α* (HIF-1*α*) [11], has been associated with increased hyaluronan accumulation [11, 12].

The hyaluronan molecule, composed of 2,000–25,000 disaccharides, with molecular weight of 1–10 million Da, is synthesized at the plasma membrane by three transmembrane synthases (HAS1-3) and is simultaneously extruded to extracellular space. Hyaluronan interacts with several binding proteins and can be incorporated into the extracellular matrix or bound to its cell surface receptors. Hyaluronan binding to its best-characterized receptor, CD44, induces activation of the PI3K-Akt and MAP kinase pathways and promotes tumor cell proliferation, invasion, and chemoresistance [13, 14]. Accumulation of intracellular hyaluronan is also found in several cell types [15–17] where it has been suggested to be a result of hyaluronan endocytosis and degradation [18] or activation of intracellular hyaluronan synthesis [17, 19]. Hyaluronan is degraded mainly by the hyaluronidase-1 (HYAL1) and hyaluronidase-2 (HYAL2) enzymes [20]. In addition to hyaluronidases, exoglycosidases and reactive oxygen species are known to participate in the degradation of high molecular weight hyaluronan to smaller fragments [21]. Recently, KIAA1199 has been suggested to be a hyaluronan binding protein and may be involved in hyaluronan degradation that is independent of CD44 and HYAL enzymes [22].

Hyaluronan synthases share 57–71% identity at the amino acid level [23] but have different expression patterns, differ in their enzymatic properties, and are differentially regulated [24]. Overexpression of any of the HAS genes in COS-1 and rat fibroblasts leads to the formation of a pericellular hyaluronan coat, where HAS1 produces smaller coats compared to HAS2 or HAS3 [25, 26]. The HAS2 isoform has been most studied and is the only isoform required for embryonic development [27]. HAS2 is believed to be the main HAS in epithelial cells and has been reported to mediate epithelial-mesenchymal transition (EMT) [28, 29]. Overexpression of HAS3 leads to the formation of long plasma membrane protrusions [30] which have recently been associated with the increased release of hyaluronan-coated and plasma membrane-derived microvesicles [31]. Moreover, overexpression of HAS3 has been reported to induce misorientation of the mitotic spindle and disturbed organization of epithelium [32], which is associated with malignancies [33]. Recently, HASs have also been suggested to form homodimers and heterodimers which may affect their function and regulate their activity [24, 34].

In human pancreatic cancer, 87% of tumors contain high levels of hyaluronan [4–6]. Similarly, extensive hyaluronan deposition is also found in a transgenic mouse model (*LSL-Kras* ^*G12D/+*^ *; LSLTrp53* ^*R172H/+*^ *;* and* Pdx-1-Cre* (KPC)) of pancreatic adenocarcinoma [4, 6]. The importance of hyaluronan in the pancreatic cancer microenvironment is further supported by the finding that pancreatic cancer cells encapsulated within hyaluronan gel produce faster growing tumors and are more metastatic than cancer cells with no hyaluronan in a mouse model [35]. Suppression of hyaluronan synthesis by downregulation of HASs has been previously shown to inhibit the growth of implanted breast, prostate, squamous cell carcinoma, and osteosarcoma tumors [15, 36–38]. Similarly, hyaluronan synthesis inhibitor 4-methylumbelliferone or its derivatives suppress metastasis of several tumor types [38–41].

In agreement with previous findings, enzymatic removal of hyaluronan by pegylated human recombinant hyaluronidase (PEGPH20) leads to suppression of tumor growth and metastasis and enhanced delivery of chemotherapy in hyaluronan-rich tumor models of prostate, lung, and pancreatic cancer [5, 9, 42]. In the KPC mouse model of pancreatic adenocarcinoma that closely resembles human disease, PEGPH20 suppressed tumor growth, increased drug delivery, and increased overall survival when used in combination with gemcitabine compared to gemcitabine monotherapy [4, 6]. Increased drug delivery of gemcitabine was associated with stromal remodeling, reduction of tumor interstitial fluid pressure, expansion of intratumoral blood vessels, and ultrastructural changes in tumor endothelium, characterized as formation of fenestrae in tumor endothelium [4, 6].

Over the years, HAS2 has been the focus of most research in this area and has been widely associated with malignant transformation and aggressive tumor growth [28, 29, 43]. However, elevated HAS3 protein levels have also been associated with ovarian cancer [44], and overexpression of HAS3 promotes tumor growth in a preclinical model [45]. Regulation and possible differential mechanisms of HAS2- and HAS3-mediated tumor growth are not completely understood. To date there are no reports comparing the roles of HAS2 and HAS3 in pancreatic cancer. In this study, we explored the biological consequences of HAS2 and HAS3 overexpression in BxPC-3 pancreatic cancer cells and in xenograft tumor models. HAS3 overexpression led to increased accumulation of extracellular hyaluronan that was associated with faster tumor growth and enhanced response to PEGPH20. Deposition of extracellular hyaluronan was associated with loss of adhesion proteins from the plasma membrane that was inhibited by hyaluronan depletion. These results are further supported by the finding that more plasma membrane E-cadherin was observed in KPC tumors as well as in a human non-small cell lung cancer (NSCLC) patient biopsy after PEGPH20 therapy.

## 2. Material and Methods

### 2.1. Cell Lines

BxPC-3 cells were obtained from American Type Culture Collection (Manassas, VA). BxPC-3 cells and lentiviral-transduced BxPC-3 cell lines overexpressing empty vector, HAS2, or HAS3 were cultured in RPMI medium with L-glutamine (Cat# 10-040-CV, Cellgro, Mediatech, Manassas, VA) containing 10% fetal bovine serum (Foundation B, Gemini Bio-Products, Sacramento, CA) at 37°C in a humidified incubator supplied with 5% CO_2_/5% air. For experimental use, cells were thawed and maintained in culture for no longer than 10 passages. Each cell line was routinely tested negative for mycoplasma contamination by MycoAlert Mycoplasma Detection Kit (Lonza Biologics, Hopkinton, MA).

### 2.2. Establishment of BxPC-3 Vector, BxPC-3 HAS2, and BxPC-3 HAS3 Cell Lines

Lentiviral vectors pLV-EF1a-MCS-IRES-Hyg (vector only), pLV-EF1a-hHAS2-IRES-Hyg (HAS2), and pLV-EF1a-hHAS3-IRES-Hyg (HAS3, Biosettia, San Diego, CA) were added to subconfluent BxPC-3 cultures, followed by centrifugation for 30 min at 1,200 rpm and incubation at 37°C for 6 h. Fresh medium was added to the cultures, and incubation was continued for 48 h, followed by selection and expansion of stable cultures using medium containing hygromycin.

### 2.3. Hyaluronidase-Sensitive Particle Exclusion Assay

To visualize aggrecan-mediated hyaluronan pericellular matrices in vitro, particle exclusion assays were performed as previously described [9, 42], with some modifications. Subconfluent cultures were treated with 1 mg/mL bovine nasal septum proteoglycan (Elastin Products, Owensville, MO) for 1 h at 37°C, followed by incubation with vehicle or 1,000 U/mL recombinant human PH20 (rHuPH20, Halozyme Therapeutics, San Diego, CA) as a negative control for 2 h at 37°C. Glutaraldehyde-fixed mouse red blood cells were added to the cultures, which were then imaged with a phase-contrast microscope coupled with a camera scanner and SPOT advanced imaging program (Version 4.6, Diagnostic Instruments, Sterling Heights, MI). Particle exclusion area and cell area were measured and relative hyaluronan coat area was calculated using the formula:* matrix area–cell area* (expressed as *μ*m^2^).

### 2.4. Hyaluronan Assay

To analyze hyaluronan secretion, tissue culture supernatants were collected from subconfluent cultures after 24 h culture, and cells were trypsinized and counted for normalization. Hyaluronan concentration in the samples was quantified using an enzyme-linked hyaluronan-binding protein sandwich assay (Cat# DY3614, R&D Systems, Minneapolis, MN) according to manufacturer's instructions [42].

### 2.5. Isolation of Secreted, Cell Surface, and Intracellular Hyaluronan

Isolation of secreted, cell surface, and intracellular hyaluronan was performed as previously described [16], with some modifications. The conditioned media containing secreted hyaluronan were collected from subconfluent cultures after 48 h of culture. Cells were detached and collected by centrifugation and supernatant was transferred to a clean tube. Cell pellets were rinsed with 1 × PBS and centrifuged, and supernatant was combined with the supernatant from the previous centrifugation and was designated as “cell surface hyaluronan.” Combined supernatants were incubated briefly at 100°C to inactivate trypsin. The cell pellet was rinsed, the supernatant was discarded, and the cell pellet was designated as “intracellular hyaluronan.” To ensure that no residual cell surface hyaluronan was present, some of the cell fractions were treated with 1,000 U/mL rHuPH20 for 20 min and rinsed with ice-cold 1 × PBS followed by centrifugation and collection of the cell pellet. All the samples were digested with Proteinase K at +55°C overnight, followed by heat inactivation at 95°C for 10 min and centrifugation at 12,000 rpm at 4°C for 10 min.

### 2.6. Fluorescent Staining of Intracellular Hyaluronan and CD44

For fluorescent staining, subconfluent cultures were fixed with 4% paraformaldehyde, and pericellular hyaluronan was digested with rHuPH20 (Halozyme Therapeutics, Inc., San Diego, CA). Cells were then permeabilized with 0.3% Triton-x-100 (Calbiochem, La Jolla, CA; 1% BSA) and blocked with 3% BSA. Hyaluronan and CD44 were stained with biotinylated hyaluronan-binding protein (bHABP, 2.5 *μ*g/mL, Calbiochem/EMD Chemicals, Inc., San Diego, CA) and anti-CD44 antibody (1 *μ*g/mL, BBA10, R&D Systems) overnight at 4°C. bHABP and anti-CD44 antibody were detected by Fluorescein-labeled streptavidin (0.5 *μ*g/mL, Vector Laboratories, Burlingame, CA) and Texas Red labeled anti-mouse antibody (0.5 *μ*g/mL, Vector), respectively. Nuclei were stained with Prolong Antifade with 4′,6-diamidino-2-phenylindole (DAPI; Invitrogen, Carlsbad, CA), and cultures were imaged with a spinning disk microscope (Nikon Eclipse TE 2000-U (Nikon, Eugene, OR) and BD Bioscience spinning disk unit (BD Bioscience, Rockville, MD)) using 60x objective and MetaMorph software version 7.7.6.0 (Molecular Devices, Sunnyvale, CA).

### 2.7. Pancreatic Cancer Xenograft Models

Six- to eight-week-old nu/nu (Ncr) athymic mice, handled in accordance with approved Institutional Animal Care and Use Committee protocols, were used for xenograft studies. Mice were inoculated with 0.05 mL of 5 × 10^6^ BxPC-3, BxPC-3 vector, BxPC-3 HAS2, or BxPC-3 HAS3 cells (concentration 1 × 10^8^ cells/mL) peritibially in the hind leg (adjacent to the tibia periosteum). Tumor growth was monitored by ultrasound imaging (VisualSonics Vevo 770/2100 High Resolution Imaging System (VisualSonics, Ontario, Canada)) until the average tumor size reached 200–500 mm^3^. The animals were then divided into treatment groups and treated intravenously (*i.v.*) twice a week for 2-3 weeks with vehicle, 37.5 *μ*g/kg, 1,000 *μ*g/kg, or 4,500 *μ*g/kg PEGPH20. At the end of the study, tumors were collected and divided into parts; one part was fixed in 10% formalin for histology, and the other parts were frozen for later biochemical analysis. Some additional tumor-bearing mice with large tumors (~2,000 mm^3^) received two injections of vehicle or 4,500 *μ*g/kg PEGPH20, and tumors were harvested in 10% formalin 6 h after treatment.

### 2.8. KPC Mouse Model

Mouse pancreatic cancer tissue sections from KPC mice [46] were obtained from the Jacobetz et al. 2013 study [4]. As described previously, mice were treated* i.v.* with a single injection of 4,500 *μ*g/kg PEGPH20 once tumor volume reached 270 mm^3^, and tumors were collected 1, 8, 24, and 72 h after dosing [4].

### 2.9. Human Patient Samples

Pretreatment and posttreatment biopsies were obtained from a patient with advanced NSCLC who was enrolled in a Phase 1 study of PEGPH20 given* i.v.* to patients with advanced solid tumors (NCT01170897). The protocol was approved by Institutional Review Board and all patients consented to study. The patient was treated with a single dose of 5.0 *μ*g/kg PEGPH20. Posttreatment biopsy was taken 7 days after* i.v.* treatment.

### 2.10. Tissue Samples

Tissues were fixed in 10% neutral buffered formalin and processed to paraffin. Five micrometer sections were used for Hematoxylin and Eosin (H&E) staining that was performed according to a standard protocol. For hyaluronan assay, fresh frozen tumor pieces were digested with Proteinase K at +55°C overnight, then heat-inactivated at 95°C for 10 min, and centrifuged at 12,000 rpm at 4°C for 10 min.

### 2.11. Hyaluronan Staining

Hyaluronan in tumor tissues was localized as previously described [42], with some modifications. Briefly, five micrometer sections were deparaffinized and rehydrated, and endogenous peroxidase was blocked with Peroxo-Block solution (Invitrogen). Nonspecific staining was blocked using 2% BSA (Jackson ImmunoResearch, West Grove, PA) and 2% normal goat serum (Vector) for 1 h, followed by blocking of endogenous avidin and biotin (Avidin/Biotin Blocking Kit, Invitrogen). Hyaluronan was detected by incubating sections with 2.5 *μ*g/mL bHABP (Seikagaku, Tokyo, Japan) for 1 h at 37°C. Signal was amplified by incubation with streptavidin-horseradish peroxidase solution (HRP; BD Biosciences) and detected with 3,3′-diaminobenzidine (DAB, Dako North America, Carpinteria, CA). Sections were then counterstained in Gill's hematoxylin (Vector) and mounted in Cytoseal 60 medium (American MasterTech, Lodi, CA). Specificity of the staining was assessed by incubation of a section of each sample with rHuPH20 (12,000 U/mL) in PIPES buffer (25 mM PIPES, 70 mM NaCl, 0.1% BSA, pH 5.5) at 37°C for 2 h prior to incubation with bHABP.

### 2.12. Immunohistochemistry

For cleaved caspase-3 (CC3), phosphohistone 3 (PH3), E-cadherin, and *β*-catenin immunohistochemistry, five micrometer sections were deparaffinized, rehydrated, and pretreated in boiling citrate buffer (pH 6.0) for 10 min. For CC3 and *β*-catenin stainings, endogenous peroxidase was blocked with 3% H_2_O_2_, followed by blocking with 5% normal goat serum. Sections were incubated with CC3 (0.21 *μ*g/mL, Cat# 9661, Cell Signaling, Danvers, MA) and *β*-catenin (40 *μ*g/mL, Cat# 8480, Cell Signaling) antibodies in SignalStain Antibody Diluent (Cell Signaling) overnight at 4°C. Signal was detected with anti-rabbit SignalStain Boost IHC Detection Reagent (Cat# 8114, Cell Signaling) and DAB. For PH3 immunohistochemistry, sections were blocked with 3% H_2_O_2_ and 5% normal goat serum. Then PH3 antibody (65 ng/mL, Cat# 9701, Cell Signaling) was added and incubated overnight at 4°C. Primary antibody was detected with HRP-conjugated anti-rabbit immunoglobulin G (IgG) (0.17 *μ*g/mL, Cat# 7074, Cell Signaling) and DAB. For E-cadherin immunohistochemistry, slides were blocked with 3% H_2_O_2_, followed by blocking of endogenous avidin and biotin. E-cadherin (0.25 *μ*g/mL, ab76055, Abcam, Cambridge, UK) antibody was diluted in mouse-on-mouse (M.O.M.) diluent and added to the sections for 1 h (Cat# PK-2200, Vector). Subsequently, M.O.M biotinylated anti-mouse IgG was added to the slides, and signal was amplified with Vectastain ABC reagent (Vector) and DAB. Then slides were counterstained with Gill Hematoxylin and mounted with synthetic media.

### 2.13. Western Blot

Tumor pieces were homogenized in cold hypotonic buffer (25 mM Tris. HCl pH 7.4, 50 mM NaCl, 10% glycerol, 0.5% Triton X-100, and 5 mM EDTA with protease and phosphatase inhibitor cocktail); the homogenates were incubated at 4°C for 30 min and centrifuged at 7,500 rpm for 10 min at 4°C. The pellet was resuspended in nuclear extraction buffer (25 mM Tris-HCl pH 7.4, 250 mM NaCl, 10% glycerol, 0.5% Triton X-100, and 5 mM MgCl_2_ with protease and phosphatase inhibitor cocktail), treated with protease-free DNAse I, briefly sonicated, and incubated with 200 *μ*g/mL ethidium bromide for 1 h. Finally, samples were centrifuged and the supernatant was collected. Nuclear extracts were separated on SDS-PAGE, transferred into a nitrocellulose membrane, and blocked with 5% BSA in PBS with 0.1% Tween 20. The membrane was probed with HIF-1*α* antibody (0.5 *μ*g/mL, ab16066, Abcam), Snail (1 *μ*g/mL, ab130245, Abcam), *β*-actin (13 *μ*g/mL, Cat# 2148, Cell Signaling), and Histone 3 (1.1 *μ*g/mL, Cat# 9717, Cell Signaling) antibodies. HIF-1*α* and Snail antibodies were detected with HRP-conjugated goat anti-mouse IgG antibody (1 : 10,000, Jackson ImmunoResearch) and chemiluminescent reagent (GE Healthcare Life Sciences, Piscataway Township, NJ) and *β*-actin and Histone 3 with HRP-conjugated anti-rabbit IgG and chemiluminescent reagent. Images were captured using GE ImageQuant 400 (GE Healthcare) and bands were quantified using Image-Pro Analyzer 7.0 software (Media Cybernetics, Rockville, MD).

### 2.14. Quantification of Staining

Stained sections were scanned at Flagship Biosciences LLC (Boulder, CO) and automated quantification of CC3 staining was performed using Aperio Positive Pixel algorithm v9 (Aperio, Buffalo Grove, IL) and results were normalized to the total number of pixels. Quantification of PH3 stainings was performed by Aperio Imagescope using Nuclear v9 algorithm.

### 2.15. Statistical Analysis

Values are presented as mean ± S.D. or SEM. Results expressed as ratios or percent values were log transformed prior to the analysis. Statistical difference was analyzed by *t* test, one-way ANOVA, and Tukey's post hoc test or two-way ANOVA with Bonferroni's post hoctest using Graphpad Prism 5 software (Graphpad, La Jolla, CA). Statistical difference was set at *P* < 0.05 (*P < 0.05; **P < 0.01; and ***P < 0.001).

## 3. Results and Discussion

### 3.1. HAS3 Overexpression Is Associated with Increased Levels of Hyaluronan Production

Extensive hyaluronan accumulation is found in pancreatic cancer [4, 6], which is characterized by massive desmoplastic stroma, high interstitial tumor pressure, poor perfusion, and resistance to therapy [47]. Hyaluronan is synthesized by three HAS enzymes at the plasma membrane, and HAS overexpression has been associated with cancer growth [43, 45, 48]. In this study, we used BxPC-3 pancreatic cancer cells that overexpress HAS3 to assess the roles of this HAS isoform on pancreatic cell lines and tumors.

Human BxPC-3 pancreatic adenocarcinoma cells were engineered to overexpress HAS3, and functional consequences of hyaluronan production by HAS3 were analyzed by hyaluronan secretion and size of pericellular hyaluronan matrix. BxPC-3 cells secreted 181 ng hyaluronan to culture medium per 10,000 cells over 24 h, while hyaluronan secretion of BxPC-3 HAS3 cells was 20-fold higher, at 3,607 ng per 10,000 cells (Table 1). Similarly, relative hyaluronan matrix area was increased by 2.4-fold after HAS3 overexpression (Table 1). Overexpression of HAS2 and HAS3 has been reported to lead to increased hyaluronan secretion and increased size of hyaluronan coat in several cell lines [25, 26]. Thus, we generated and tested HAS2-overexpressing BxPC-3 cells and found that similar amounts of hyaluronan were secreted by BxPC-3 HAS2 and BxPC-3 HAS3 cells (Table 1), resulting in similar hyaluronan coat sizes. The size of hyaluronan coat on BxPC-3 cells transduced with an empty vector was also analyzed, and coat size did not significantly differ from that on parental cells (data not shown).

**Figure 1:**
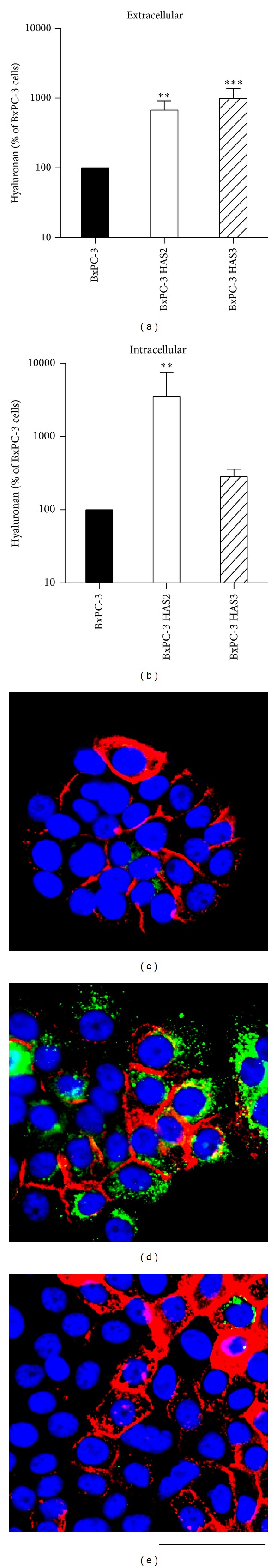
HAS3 overexpression is associated with high extracellular hyaluronan accumulation and HAS2 with increased extracellular and intracellular accumulation of hyaluronan. Amount of secreted, cell surface, and intracellular hyaluronan was analyzed in BxPC-3, BxPC-3 HAS2, and BxPC-3 HAS3 cells with a hyaluronan assay, and results were presented as extracellular hyaluronan showing combined amount of secreted and cell surface hyaluronan (a) and intracellular hyaluronan (b). The results were expressed as % of hyaluronan content in BxPC-3 cells, and data represent mean ± S.D. of three independent experiments. To visualize intracellular hyaluronan, subconfluent cultures of BxPC-3 (c), BxPC-3 HAS2 (d), and BxPC-3 HAS3 cells (e) were stained for intracellular hyaluronan (green) and CD44 (red) using bHABP and anti-CD44s-antibody, respectively. Nuclei were visualized with Prolong Antifade with DAPI. Scale bar in (c–e) is 50 *μ*m. Statistical differences between the groups in (a) and (b) were tested using one-way ANOVA and Tukey's post hoc test (**P < 0.01; ***P < 0.001).

To further characterize BxPC-3 HAS3 cells, the quantity of extracellular and intracellular hyaluronan was analyzed. In line with earlier results (Table 1), the amount of extracellular hyaluronan, composed of secreted and cell surface hyaluronan, was 9.9-fold higher in BxPC-3 HAS3 cells compared to BxPC-3 cells (Figure 1(a)). Overexpression of HAS3 also induced a slight 2.8-fold increase in intracellular hyaluronan content (Figure 1(b)). HAS2-overexpressing cells contained a similar amount of extracellular hyaluronan as HAS3-overexpressing cells (Figure 1(a)), but their intracellular hyaluronan level was 35-fold higher than in BxPC-3 cells (Figure 1(b)). Intracellular hyaluronan was also visualized in cultures after fixation and digestion of cell surface hyaluronan. CD44 was stained in the cultures to visualize plasma membranes of the cells. Parental or HAS3-overexpressing cells contained very little intracellular hyaluronan (Figures 1(c) and 1(e)) while HAS2-overexpressing cells showed intensive accumulation of intracellular hyaluronan (Figure 1(d)). These data are consistent with previous reports that HAS2 expression has been associated with the presence of intracellular hyaluronan, induced by epidermal growth factor, keratinocyte growth factor, or hyperglycemia [16, 19, 49].

### 3.2. Amount and Localization of Hyaluronan in HAS3-Overexpressing Xenograft Tumors

**Figure 2:**
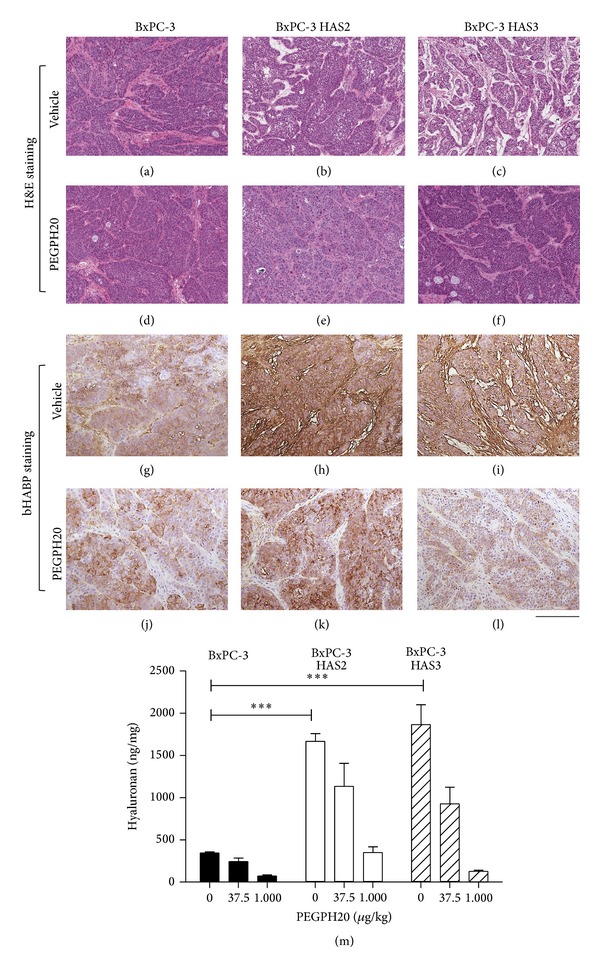
Localization and amount of hyaluronan in BxPC-3, BxPC-3 HAS2, and BxPC-3 HAS3 tumors and response to PEGPH20. Mice carrying BxPC-3, BxPC-3 HAS2, and BxPC-3 HAS3 peritibial xenograft tumors were treated twice with vehicle or 4,500 *μ*g/kg PEGPH20, and tumors were collected 6 h after the last dose. Tumor sections were stained with H&E to visualize morphology of the tumors (a–f) and with bHABP for hyaluronan (g–l). Scale bar in (a–f) and (g–l) is 500 *μ*m. Hyaluronan concentration in the tumors after two weekly treatments of vehicle, 37.5 *μ*g/kg or 1,000 *μ*g/kg of PEGPH20 (*n* ≥ 3/group) for three weeks was analyzed by hyaluronan assay (m). Statistical differences between the groups shown in figure (m) were tested using two-way ANOVA and Bonferroni's post hoc test (*P < 0.05; **P < 0.01; ***P < 0.001).

In a follow-up in vivo study, mice bearing BxPC-3 and BxPC-3 HAS3 tumors were dosed twice with vehicle or 4,500 *μ*g/kg PEGPH20, and tumors were collected 6 h after the second dosing. Histochemistry of the tumors revealed that HAS3 overexpression changed the structure of the tumor stroma (Figures 2(a)–2(c)). HAS3-overexpressing tumors contained a large amount of hyaluronan located in the extracellular space of the stroma, while hyaluronan in BxPC-3 tumors was mostly associated with cancer cells (Figures 2(g) and 2(i)). Total hyaluronan content of BxPC-3 HAS3 tumors was 1,864 ng/mg, while hyaluronan content of BxPC-3 tumors was 344 ng/mg (Figure 2(m)). In line with the in vitro results, BxPC-3 HAS2 tumors contained a similar amount of hyaluronan as BxPC-3 HAS3 tumors (Figures 2(h)-2(i) and 2(m)) but in addition to stromal hyaluronan accumulation, cancer cell-associated hyaluronan resembling intracellular hyaluronan was found (Figure 2(h)). Chronic treatment with PEGPH20 (twice a week dosing for 3 weeks) depleted 95% of hyaluronan in BxPC-3 HAS3 tumors, while in BxPC-3 and BxPC-3 HAS2 tumors hyaluronan removal was 72% (Figure 2(m)). The results are consistent with previous reports showing that PEGPH20 can efficiently remove hyaluronan in the tumors [4, 6, 9, 42]. Hyaluronan staining of the PEGPH20-treated tumors revealed that PEGPH20 removed most of the extracellular hyaluronan (Figures 2(j)–2(l)) and reduced the amount of stroma (Figures 2(d)–2(f)), although intracellular hyaluronan in cancer cells was still apparent (Figures 2(j)–2(l)). After treatment with PEGPH20, intracellular hyaluronan was most prominent in HAS2 tumors (Figure 2(k)), in agreement with in vitro observation of intracellular accumulation of hyaluronan in HAS2-overexpressing cells (Figures 1(b) and 1(d)). The significance and origin of intracellular hyaluronan accumulation in HAS2-overexpressing pancreatic cancer cells and tumors is a subject requiring further investigation. Coexpression of HAS2 with CD44 and HYAL2 has been reported and could potentially lead to the cleavage of hyaluronan at the plasma membrane and CD44-mediated internalization and accumulation of intracellular hyaluronan [36, 50, 51]. Intracellular hyaluronan synthesis has also been reported in hyperglycemia and inflammatory conditions [17, 19]. These results also confirm that PEGPH20 only degrades hyaluronan within the extracellular space, probably due to lack of a mechanism to enter the cells.

### 3.3. HAS3 Overexpression in BxPC-3 Cells Is Associated with Faster In Vivo Growth and Enhanced Tumor Growth Inhibition

**Figure 3:**
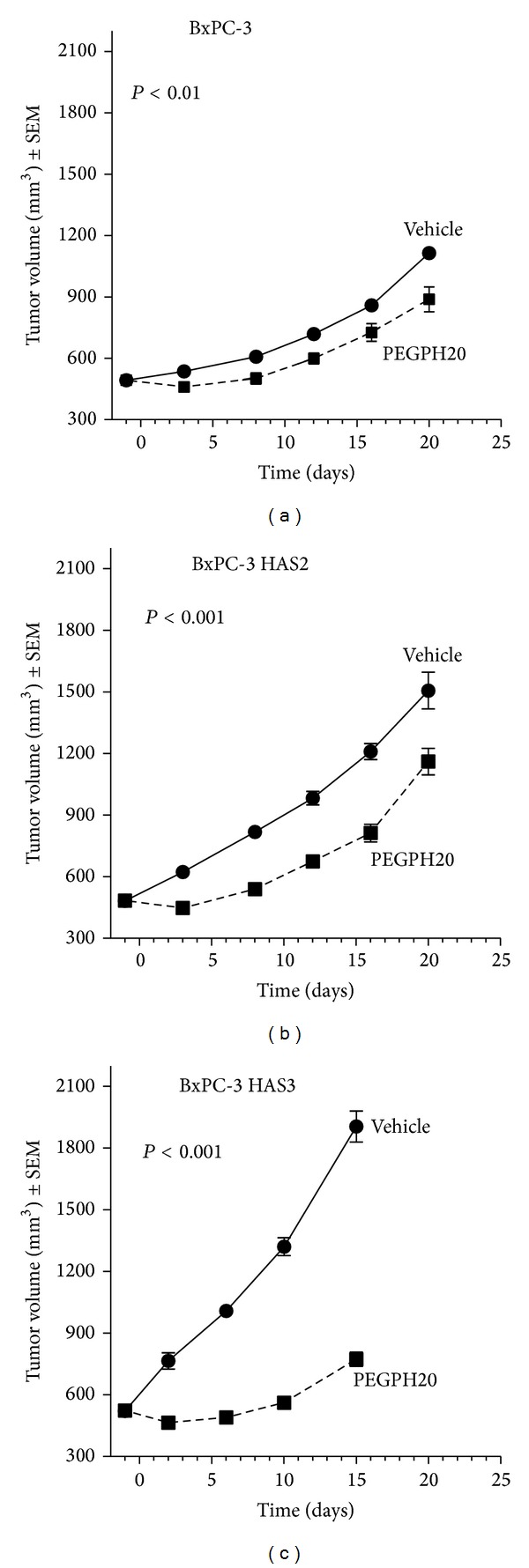
Pancreatic cancer xenograft tumors overexpressing HAS3 grow faster and respond better to hyaluronan removal than HAS2 or parental tumors. To compare in vivo growth, BxPC-3, BxPC-3 HAS2, and BxPC-3 HAS3 tumor cells were inoculated adjacent to the tibial periosteum in the hind limb of nu/nu mice (*n* = 7/group), and tumor growth was monitored using ultrasound imaging. Once average tumor size reached 500 mm^3^, mice were treated twice a week with an* i.v.* injection of vehicle or PEGPH20 (4,500 *μ*g/kg) (a–c). Statistical difference was tested using repeated measured two-way ANOVA and Bonferroni's post hoc test.

HAS2 has been widely shown to be associated with cancer [43, 48]; however, much less is known about the role of HAS3 in tumor progression. To compare the in vivo growth rate and PEGPH20 response of BxPC-3 and BxPC-3 HAS3 cells, both cells were inoculated peritibially in the hind limb of nude mice to generate tumors, and the mice were dosed* i.v.* twice a week with vehicle or 4,500 *μ*g/kg PEGPH20. BxPC-3 HAS3 cells generated faster growing tumors than parental cells (Figures 3(a) and 3(c)), which correlates with the amount of extracellular hyaluronan in the tumor stroma (Figures 2(g) and 2(i)). The average size of BxPC-3 HAS3 tumors was 1,905 mm^3^ on day 15 after initiation of vehicle treatment while the average size of BxPC-3 tumors was 820 mm^3^ (Figures 3(a) and 3(c)). This is consistent with the report that HAS3 overexpression enhances extracellular matrix deposition and tumor growth of prostate cancer cells [45] and that metastatic colon cancer cells have upregulated levels of HAS3 [52]. HAS3 protein level is higher in human ovarian cancer than in normal ovary, and the amount of HAS3-positive cancer cells correlates with hyaluronan accumulation in the stroma [44]. Overexpression of HAS2 also increased the tumor growth in a BxPC-3 model but led to less aggressive tumors than those with HAS3 overexpression (Figure 3(b)). Stromal hyaluronan has been reported to contribute to high interstitial fluid pressure, compression of tumor blood vessels, and poor drug delivery in pancreatic cancer tumors [4, 6]. To date there have been no observed substantial differences in the amount of stromal hyaluronan between HAS2- and HAS3-overexpressing tumors. However, consequences of HAS2 and HAS3 overexpression on vasculature are not well understood and the possibility that HAS2 and HAS3 might have distinct effects on vascular function cannot be completely ruled out. Interestingly, BxPC-3 HAS3 tumors showed a strong response to PEGPH20, which caused 86% tumor growth inhibition (Figure 3(c)). In BxPC-3 and BxPC-3 HAS2 tumors, PEGPH20 induced tumor growth inhibition of 34% and 32%, respectively (Figures 3(a) and 3(b)). Removal of hyaluronan by PEGPH20 has been shown to lead to decompression of tumor blood vessels, changed ultrastructure of tumor endothelia, and increased drug delivery to the tumor [4, 6, 9]. We show that intravenous injection of PEGPH20 led to similar depletion of stromal hyaluronan in HAS2- and HAS3-overexpressing tumor models (Figures 2(k) and 2(l)) suggesting no differences in drug delivery of PEGPH20 to these tumor types. Taken together, the data show that tumors with HAS3 overexpression show more aggressive tumor growth than parental tumors or tumors with HAS2 overexpression. In HAS3-overexpressing tumors, PEGPH20 removes most of the hyaluronan and has a strong inhibitory effect on tumor growth, whereas in HAS2-overexpressing tumors PEGPH20 removes the extracellular but not the intracellular hyaluronan and causes a weaker inhibitory effect on tumor growth. Our results are in line with previous work showing that depletion of hyaluronan or suppression of its synthesis leads to inhibition of tumor growth in multiple tumor models [9, 36–38, 41, 42].

### 3.4. Accumulation of Extracellular Hyaluronan by HAS3 Overexpression Induces Loss of Plasma Membrane E-Cadherin

**Figure 4:**
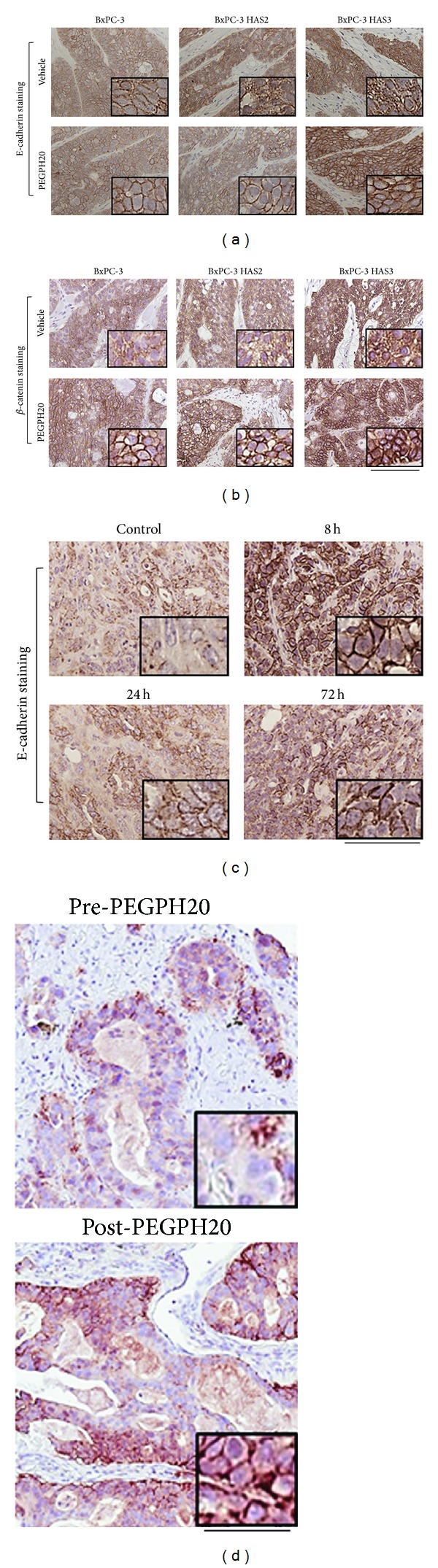
HAS overexpression induces loss of plasma membrane E-cadherin and accumulation of cytoplasmic *β*-catenin, and removal of hyaluronan translocates them to the plasma membrane. Vehicle- and PEGPH20- (2 doses; 4,500 *μ*g/kg; *n* = 3/group) treated tumors were stained for E-cadherin (a) and *β*-catenin (b). E-cadherin was also localized in pancreatic tumors from a KPC mouse model after 0, 8, 24, and 72 h treatment with PEGPH20 (c; *n* = 3/group) and in human NSCLC biopsies before and after PEGPH20 therapy (d). Scale bar in (a–d) is 250 *μ*m. Insets in (a–d) represent 3x magnification of the original micrograph.

Hyaluronan is known to be involved in EMT-associated changes in cancer progression [27–29]. E-cadherin and *β*-catenin are essential adhesion molecules in normal epithelium, and loss of plasma membrane E-cadherin and catenins leading to disruption of cell-cell junctions is acknowledged to be an early indication of EMT [53, 54]. Because accumulation of hyaluronan by HAS3 in intercellular space has been suggested to interrupt cell-cell interactions [55], we hypothesized that hyaluronan removal would reverse changes that may have occurred in adhesion events. E-cadherin and *β*-catenin were visualized by immunohistochemistry in vehicle-treated and PEGPH20-treated BxPC-3 and BxPC-3 HAS3 tumors. HAS3-induced extracellular hyaluronan accumulation resulted in loss of plasma membrane E-cadherin (Figure 4(a)) and increased accumulation of cytoplasmic *β*-catenin in tumor cells of BxPC-3 tumors (Figure 4(b)). This result supports previous findings that increased hyaluronan production leads to disruption of adherens junctions and leads to EMT [28, 43]. Since the majority of pancreatic adenocarcinomas have high hyaluronan accumulation [4, 5]; these results are in line with the previously reported loss of membrane E-cadherin in human pancreatic adenocarcinoma compared to pancreatic intraepithelial neoplasia or normal ducts [53]. Additionally, HAS overexpression also induces loss of plasma membrane E-cadherin in breast cancer cells and spongiotic keratinocytes [28, 43, 55]. Overexpression of HAS2 led to the same changes in E-cadherin and *β*-catenin adhesion proteins in BxPC-3 tumors (Figures 4(a) and 4(b)). Interestingly, in mammary epithelial cells, TGF-*β*-induced EMT is mediated by HAS2 but not by extracellular hyaluronan [29]. This suggests that HAS2 and HAS3 may mediate EMT-associated events via different mechanisms. HAS3 seems to initiate EMT-associated events by extracellular hyaluronan, while the effect of HAS2 on EMT may be additionally mediated by intracellular hyaluronan accumulation or by HAS2 function that is not dependent on hyaluronan synthesis [29].

We then tested the effect of hyaluronan removal by PEGPH20 on EMT-related events in HAS3-overexpressing tumors. Removal of extracellular hyaluronan by PEGPH20 induced translocation of E-cadherin and *β*-catenin back to the plasma membrane (Figures 4(a) and 4(b)), and PEGPH20 showed a stronger effect in HAS3-overexpressing tumors than in BxPC-3 tumors. It has been previously shown that PEGPH20 removes most tumor-associated hyaluronan in the KPC mouse model [4], so we investigated translocation of E-cadherin in tumors from KPC mice treated with PEGPH20. Translocation of E-cadherin to plasma membrane was observed 8 h after treatment with PEGPH20, and it was most prominent in peripheral duct-like structures at the edges of the tumor (Figure 4(c)). However, the effect seems to be transient, since 24 to 72 h later the plasma membrane residence of E-cadherin started to disappear (Figure 4(c)). E-cadherin status was also studied in pretreatment and posttreatment biopsies of a lung cancer patient treated with PEGPH20 (Phase 1 study, NCT01170897). In the posttreatment biopsy, a decrease in extracellular hyaluronan (data not shown) and an increase in cell surface E-cadherin were found in comparison to the pretreatment biopsy (Figure 4(d)). Removal of extracellular hyaluronan is associated with translocation of epithelial markers E-cadherin and *β*-catenin back to the plasma membrane, suggesting a reorganization of intercellular junctions and an inhibition of early EMT-associated events, potentially contributing to growth inhibition by PEGPH20 [43].

### 3.5. Hyaluronan Removal Decreases Nuclear Levels of Hypoxia-Related Proteins

**Figure 5:**
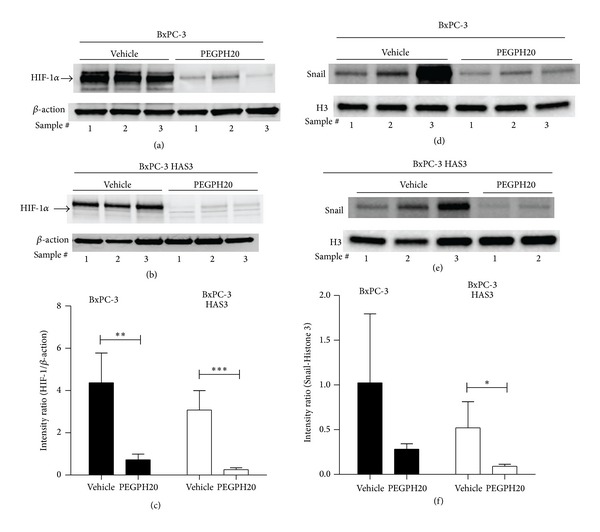
Removal of extracellular hyaluronan decreases HIF-1*α* and Snail signaling. Vehicle- and PEGPH20- (2 doses; 4,500 *μ*g/kg) treated BxPC-3 and BxPC-3 HAS3 tumors were homogenized and nuclear extracts were analyzed with Western blot using HIF-1*α* (a) and (b), Snail (d) and (e), *β*-actin (a) and (b), and Histone 3 (c) and (d) antibodies. In (a) and (b), the blot is a representative of three experiments, and each lane represents a piece of the same vehicle-treated or PEGPH20-treated tumor. The HIF-1*α* band is indicated with an arrow. In (d) and (e), lysates from the same vehicle-treated or PEGPH20-treated tumor were combined and each lane represents Snail level of one tumor. Intensities of the bands in the cropped blots were quantified using Image-Pro Analyzer 7.0 software and normalized to the intensity of the housekeeping protein ((c) and (f)). Data were plotted as mean ± S.D., and statistical difference between the groups was tested with *t* test (*P < 0.05; **P < 0.01; and ***P < 0.001).

Since hypoxia has been reported to be associated with and able to induce EMT [56, 57], we explored the effect of extracellular hyaluronan accumulation and PEGPH20 on hypoxia-related proteins in BxPC-3 tumors. Nuclear protein levels of HIF-1*α* and Snail were analyzed by Western blotting (Figures 5(a)–5(f)). Overexpression of HAS3 did not cause a major change in nuclear HIF-1*α* level, probably because BxPC-3 tumors already contain a low amount of hyaluronan (Figures 5(a)–5(c)). However, hyaluronan depletion by PEGPH20 decreases nuclear HIF-1*α* levels in both BxPC-3 and BxPC-3 HAS3 tumors (Figures 5(a)–5(c)), suggesting a decrease in hypoxic conditions after hyaluronan removal. Nuclear levels of transcription factor Snail, a target of HIF-1*α*, were also suppressed by hyaluronan depletion in HAS3-overexpressing tumors (Figures 5(d)–5(f)). These results suggest that PEGPH20 suppresses HIF-1*α*-Snail signaling in tumors with high levels of hyaluronan. In agreement with our observations, previous reports have described an association of hyaluronan accumulation and tumor hypoxia [12], and that depletion of hyaluronan synthesis by 4-methylumbelliferone prevents EMT-associated changes [58]. EMT has been reported to be mediated via HIF-1*α* signaling [56, 57], and our data suggest that removal of hyaluronan can be one step in the inhibition of this process.

### 3.6. Extracellular Hyaluronan Inhibits Apoptosis of BxPC-3 Tumors

**Figure 6:**
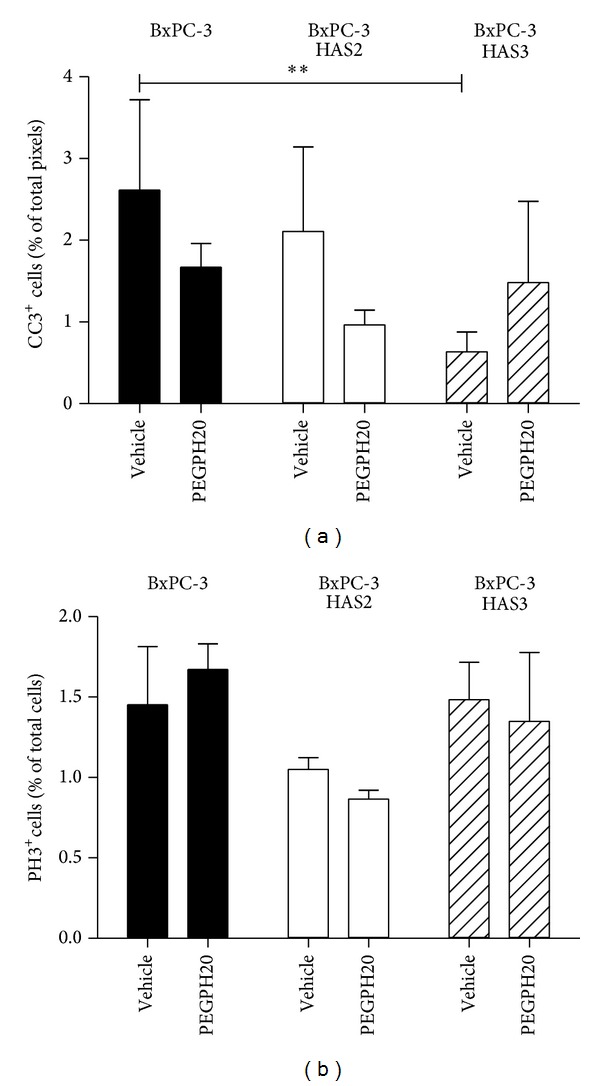
Effect of HAS2 and HAS3 overexpression and hyaluronan removal on apoptosis and proliferation in BxPC-3, BxPC-3 HAS2, and BxPC-3 HAS3 tumors. Vehicle-treated and PEGPH20-treated (2 doses; 4,500 *μ*g/kg; *n* = 3/group) BxPC-3, BxPC-3 HAS2, and BxPC-3 HAS3 xenograft tumor sections were stained for CC3 and PH3 to visualize apoptotic and proliferative cells, respectively. Number of positive cells per tumor section was analyzed using Aperio Positive Pixel v9 and Nuclear staining algorithms, respectively, ((a) and (b)). Data were plotted as mean ± S.D., and statistical difference between the groups was tested with two-way ANOVA and Bonferroni's post hoc test (**P < 0.01).

To further study the mechanism of how HAS3 overexpression favors tumor growth and leads to stronger response to PEGPH20, the amount of proliferative and apoptotic cells was analyzed in BxPC-3 and BxPC-3 HAS3 tumors. The number of apoptotic cells, assessed by CC3 positivity, was decreased in HAS3-overexpressing tumors (4-fold, *P* < 0.05), suggesting that BxPC-3 HAS3 overexpression protects cells from apoptosis (Figure 6(a)). PEGPH20 treatment showed a trend (2.3-fold) to increase CC3-positive cells in BxPC-3 HAS3 tumors but had no effect in BxPC-3 tumors (Figure 6(a)). The modest effect of PEGPH20 on promoting apoptosis in the BxPC-3 HAS3 tumor model may be due to incomplete removal of hyaluronan from the cancer cell surface (Figure 2(l)). Since HAS3 continuously produces new hyaluronan chains at the plasma membrane, binding of newly synthesized chains to hyaluronan receptors on the cancer cell surface may induce some cell survival signaling and reduce the biological effect of PEGPH20. Alternatively, overexpression of HAS3 may have other hyaluronan-independent effects on cell survival, as reported with HAS2 on TGF-*β*-induced EMT in mammary epithelial cells [29].

Cell proliferation, assessed by PH3, was not increased by HAS3 overexpression or with PEGPH20 treatment (Figure 6(b)). Overexpression of HAS2 and PEGPH20 treatment in HAS2-overexpressing tumors did not show a major effect on the number of CC3- and PH3-positive cells. These results suggest that, in addition to effects on stromal remodeling and early EMT-associated changes, accumulation of extracellular hyaluronan after HAS3 overexpression may protect pancreatic cancer cells from apoptosis. Hyaluronan removal alone may not be sufficient to induce substantial cell death in this model, but PEGPH20 may be effective in combination with chemotherapy as reported previously in the prostate cancer model [9] and in the transgenic mouse model of pancreatic cancer [6]. Consistent with our work, hyaluronan has also been previously associated with cell survival and protection of apoptosis in colon carcinoma cells [59]. In conditional Has2 transgenic mice that develop mammary tumors, Has2 overexpression decreases apoptosis but also increases the proliferation of neu-initiated tumors [43]. Differential results on proliferation may be explained by the fact that the net effect of HASs and hyaluronan on proliferation depends on several factors including cell type, cell density, and the final concentration of hyaluronan around the cells [15, 45, 60]. In KPC mice, PEGPH20 decreases proliferation and increases apoptosis in combination with gemcitabine compared to gemcitabine alone [4, 6]. Although PEGPH20 alone might not have strong effects on proliferation and apoptosis, it causes remodeling of the tumor microenvironment and could sensitize tumors to chemotherapy. The results from this study shed a light on potential application of extracellular hyaluronan and HAS3 protein expression as an indication for stromal hyaluronan depletion by PEGPH20.

## 4. Conclusions

In recent years, the importance of the tumor microenvironment in cancer progression has been increasingly recognized, and stromal components including hyaluronan have become attractive targets for cancer therapy. Hyaluronan is a major component in the stroma of many solid tumors [5, 8], and its accumulation is known to promote malignant transformation and tumor growth [28, 43, 61]. The most extensive hyaluronan accumulation is found in pancreatic cancer [4, 6], and enzymatic depletion of hyaluronan in combination with chemotherapy is currently being investigated in patients with advanced pancreatic adenocarcinoma. However, there is very little information about mechanisms leading to hyaluronan accumulation in desmoplastic response of pancreatic cancer and the roles of different HASs in these dramatic changes of tumor microenvironment.

In this study, we demonstrate that overexpression of HAS3 in pancreatic cancer cells results in more aggressive tumors with extracellular hyaluronan accumulation, whereas overexpression of HAS2 is associated with moderate-growing tumors and both extracellular and intracellular accumulations of hyaluronan. An increase in extracellular hyaluronan induces loss of plasma membrane E-cadherin and accumulation of cytoplasmic *β*-catenin, indicating disruption of epithelial cell adhesion and an early stage of EMT. PEGPH20 depletes extracellular hyaluronan and leads to strong inhibition of tumor growth in HAS3-overexpressing tumors. Tumor growth inhibition is associated with decreased nuclear levels of hypoxia-related proteins and translocation of E-cadherin and *β*-catenin to the plasma membrane. The fact that removal of hyaluronan also causes E-cadherin translocation to the plasma membrane in the transgenic mouse model and in a hyaluronan-rich human NSCLC biopsy sample further justifies the relevance of the hyaluronan-rich tumor model and highlights the role of extracellular hyaluronan in the early EMT-associated events.

Further knowledge about the mechanisms and consequences of hyaluronan accumulation in pancreatic cancer and effects of hyaluronan removal by PEGPH20 will increase our understanding of the role of hyaluronan in the development of malignancies and will enable the development of new biomarkers and therapies.

## Figures and Tables

**Table 1:** Hyaluronan production of BxPC-3, BxPC-3 HAS2, and BxPC-3 HAS3 cells.

Tumor cell line	Relative hyaluronan coat size (*μ*m^2^)^1^	Hyaluronan in CM (ng/10,000 cells in 24 h)^2^
BxPC-3	502 ± 259	181 ± 54
BxPC-3 HAS2	1417 ± 614	2832 ± 643
BxPC-3 HAS3	1154 ± 439	3607 ± 349

^1^Assessed via particle exclusion assay as described in Section 2, Mean ± S.D.

^2^Mean in culture media (*n* = 3, independent cultures) ± S.D.

## Nonstandard Abbreviations Used

bHABP: Biotinylated hyaluronan binding protein
CC3: Cleaved caspase-3
DAB: 3,3′-Diaminobenzidine
DAPI: 4′,6-Diamidino-2-phenylindole
EMT: Epithelial-mesenchymal transition
H&E: Hematoxylin and Eosin
HAS: Hyaluronan synthase
HIF: Hypoxia-inducible factor
HYAL: Hyaluronidase
IgG: Immunoglobulin G
KPC: *LSL-Kras* ^*G12D/+*^ *; LSLTrp53* ^*R172H/+*^ *; Pdx-1-Cre*
M.O.M.: Mouse-on-mouse
NSCLC: Non-small cell lung cancer
PEGPH20: Pegylated recombinant human PH20 hyaluronidase
PH3: Phosphohistone 3.
